# Supplementary material for: A set of multi-entry identification keys to African frugivorous flies (Diptera, Tephritidae)
Source: Zookeys. 2014 Jul 24;(428):97–108. doi: 10.3897/zookeys.428.7366 (PMC4143993; doi:10.3897/zookeys.428.7366)
Supplement: Supplementary material 4 — Key to Capparimyia [file zookeys-428-097-s004.zip › SF4_ZooKeys_key to Capparimyia/lucid_player/help/help/about_the_lucid_family_of_products.htm]

About the Lucid Family of Products


# About the Lucid Family of Products

The Lucid family of products comprises a series of programs to help
build, deploy and use interactive, computer-based identification keys.
Lucid keys may be used to identify a wide range of objects or entities,
from plants and animals to diseases and rocks. Usually, an expert on a
particular subject uses a Lucid program to build an identification or
diagnostic key to the species or objects of interest to them � essentially
capturing their expert knowledge in the Lucid key. The key can then be
distributed, allowing other interested people to identify and learn about
the things in the key.

The two main programs in the Lucid family are the Lucid Matrix and Lucid
Phoenix systems. Lucid3 is the latest implementation of the Lucid Matrix
system. Lucid Phoenix is used to convert existing dichotomous or branching
keys printed in books or monographs into interactive keys or to create
and deploy new pathway keys. In addition, the Lucid family includes a
number of tools designed to support users of the Lucid3 and Lucid Phoenix
systems. For more information on other members of the Lucid family of
products, please visit the Lucidcentral website at www.lucidcentral.org.
